# Seroprevalence of hepatitis D among hepatitis B-infected patients attending the viral hepatitis treatment unit in Alexandria, Egypt: a prospective cross-sectional observational study

**DOI:** 10.1186/s12879-026-13956-6

**Published:** 2026-08-14

**Authors:** Amany Elyamany, Rasha Ghazala, Mohamed Aboud, Ahmed Kamal

**Affiliations:** 1 Hepatology Unit, Internal Medicine Department, Faculty of Medicine, Alexandria University, 1 Khartoum Square, Alexandria, 21131 Egypt; 2 Medical Biochemistry Department, Faculty of Medicine, Alexandria University, Alexandria, Egypt

**Keywords:** Hepatitis D virus, Hepatitis B virus, Prevalence, Alexandria, Universal screening

## Abstract

**Background:**

Hepatitis D virus (HDV) infection accelerates liver illness progression in hepatitis B surface antigen (HBsAg)-positive individuals, yet its prevalence in Alexandria, Egypt, is unknown.

**Methods:**

We carried out a prospective cross-sectional observational study on 158 HBsAg-positive adults attending the Alexandria University Viral Hepatitis Treatment Unit (Sept 2022–Aug 2023). All underwent clinical, laboratory, and imaging assessment, and serum total anti-HDV antibodies were detected by ELISA.

**Results:**

Anti-HDV antibodies were detected in 19.6% (31/158) of patients (95% CI, 13.4%-26.8%). HDV-positive patients were older (mean 50.9 ± 12.0 vs. 45.3 ± 11.6 years, *p* = 0.024), had higher ALT (median 38 vs. 32 U/L, *p* = 0.010) and AST (median 40 vs. 31 U/L, *p* = 0.009), more frequent significant fibrosis (74.2% vs. 51.2%, *p* = 0.021), and lower HBV DNA levels (median 0.04 vs. 0.70 × 10³ IU/mL, *p* = 0.017). Hepatic encephalopathy was more common across HDV-positive participants (12.9% vs. 1.6%, *p* = 0.014).

**Conclusion:**

Nearly one in five HBsAg-positive patients in Alexandria, Egypt, had evidence of HDV exposure, with associations suggesting more advanced liver injury. These findings support universal HDV screening among all HBsAg-positive individuals in Egypt.

**Clinical trial number:**

Not applicable.

**Graphical Abstract:**

**Figure:**
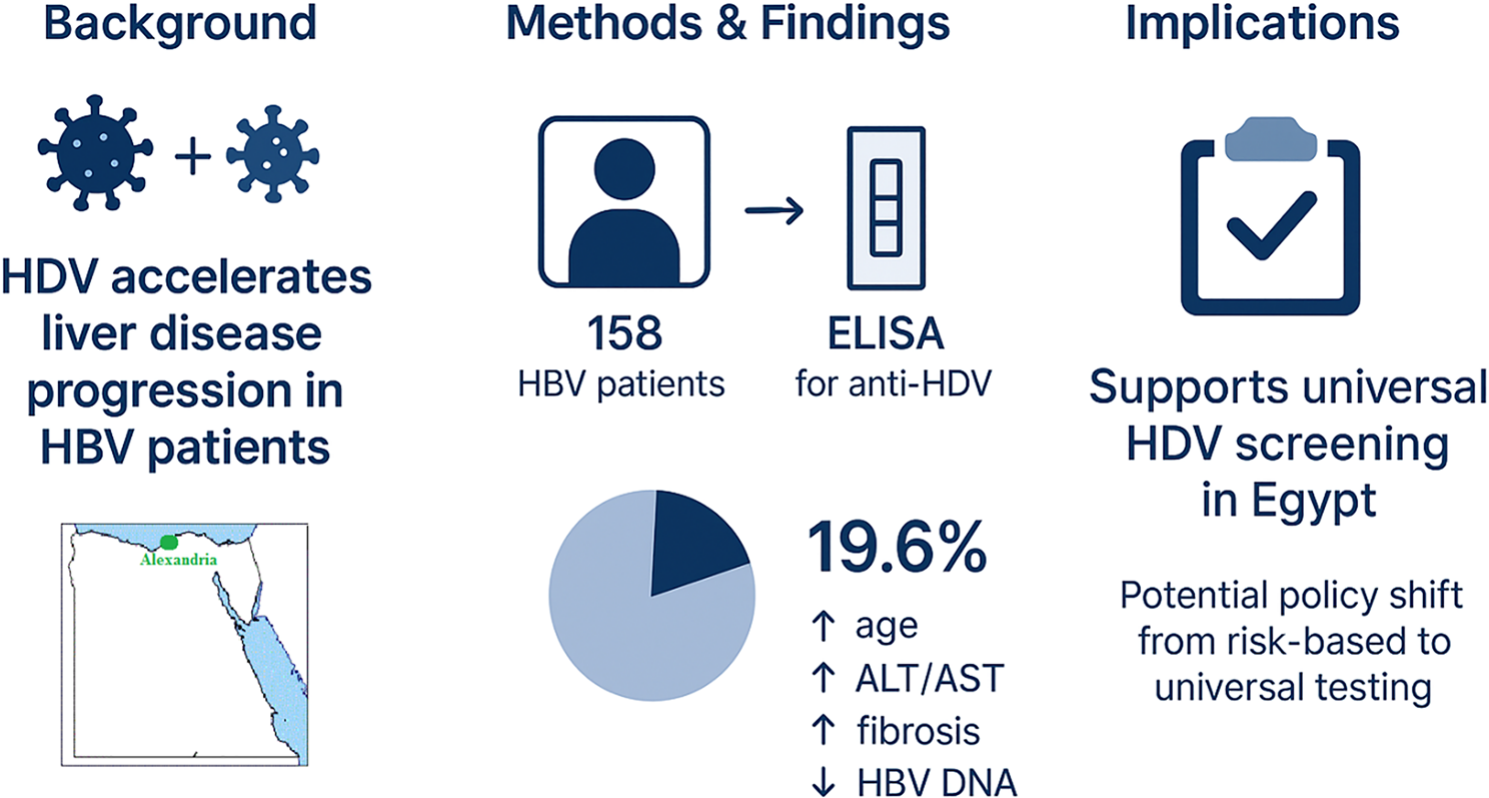

**Supplementary Information:**

The online version contains supplementary material available at 10.1186/s12879-026-13956-6.

## Introduction

Hepatitis D virus (HDV) is a satellite virus, as it necessitates hepatitis B surface antigen (HBsAg) existence for its life cycle [1]. There are 2 distinct types of HDV infection: co-infection as well as superinfection. Co-infection means the concurrent infection of both hepatitis B virus (HBV) and HDV and typically manifests as acute hepatitis and an elevated potential for acute liver failure. HBV/HDV co-infection is often temporary and self-limited, and the infection persists in approximately 5% of cases. Conversely, HDV superinfection of a person chronically infected with HBV typically results in acute hepatitis that progresses to chronicity in up to 90% of patients [2]. Chronic hepatitis D (CHD) infection leads to rapid advancement to cirrhosis and early decompensation in cirrhotic patients. Among 70% of individuals with chronic HDV superinfection, cirrhosis manifested five to ten years after initial exposure and may manifest as soon as 2 years after exposure. This represents a 3-fold escalation in disease progression in comparison with individuals infected only with HBV [3–5]. Numerous studies have also revealed a higher risk of hepatocellular carcinoma (HCC) among people co-infected with HDV in comparison to those infected only with HBV [6–8].

Three sizeable meta-analyses concerning the worldwide prevalence of HDV have been declared. The latest one, which involved 282 studies from 95 countries, revealed that the global burden of anti-HDV antibodies was 4.5% among people positive for HBsAg and 16.4% among patients visiting hepatology clinics, resulting in an estimated 12 million persons positive for anti-HDV worldwide. Furthermore, 18% of HBsAg-positive individuals with liver cirrhosis as well as 20% of those with HCC were infected with HDV. A previous meta-analysis revealed that the global prevalence of HDV was 13% within HBsAg-positive individuals, with 48–60 million persons positive for anti-HDV worldwide. Furthermore, 25% of HBsAg-positive individuals with liver cirrhosis as well as 20% of those with HCC were infected with HDV. Lastly, a third meta-analysis exploring the worldwide burden of HDV infection from 1977 to 2016 found that HDV prevalence was 14.57% among HBsAg-positive individuals, 37.57% in people who inject drugs (PWID), and 17% in individuals with risky sexual behavior, resulting in an estimated 62–72 million persons positive for anti-HDV worldwide. Nonetheless, due to the significant bias present in most studies and the inadequate screening for HDV among HBV carriers, there is considerable ambiguity in HDV prevalence estimates [9–11]. There is a gap in literature regarding HDV prevalence in different areas of Egypt, so we aimed primarily to estimate the prevalence of total anti-HDV antibodies in HBV-infected individuals who are treated at Alexandria University Hospital. The secondary aim was to determine the characteristics of anti-HDV positive patients.

## Patients and methods

### Patients

The current study is a prospective cross-sectional observational one that included 158 HBsAg-positive adults who were recruited from the Alexandria University Viral Hepatitis Treatment Unit between September 2022 and August 2023.

Based on a study by El Zayadi et al. [12] who reported a HDV seroprevalence of 37% among Egyptian patients, a sample size of 158 participants was needed to estimate the prevalence of HDV antibodies in HBsAg-positive patients with a 95% confidence level and a margin of error of 7.5%. Sample size was calculated using the NCSS 2004 and PASS 2000 programs.

**Fig. 1:**
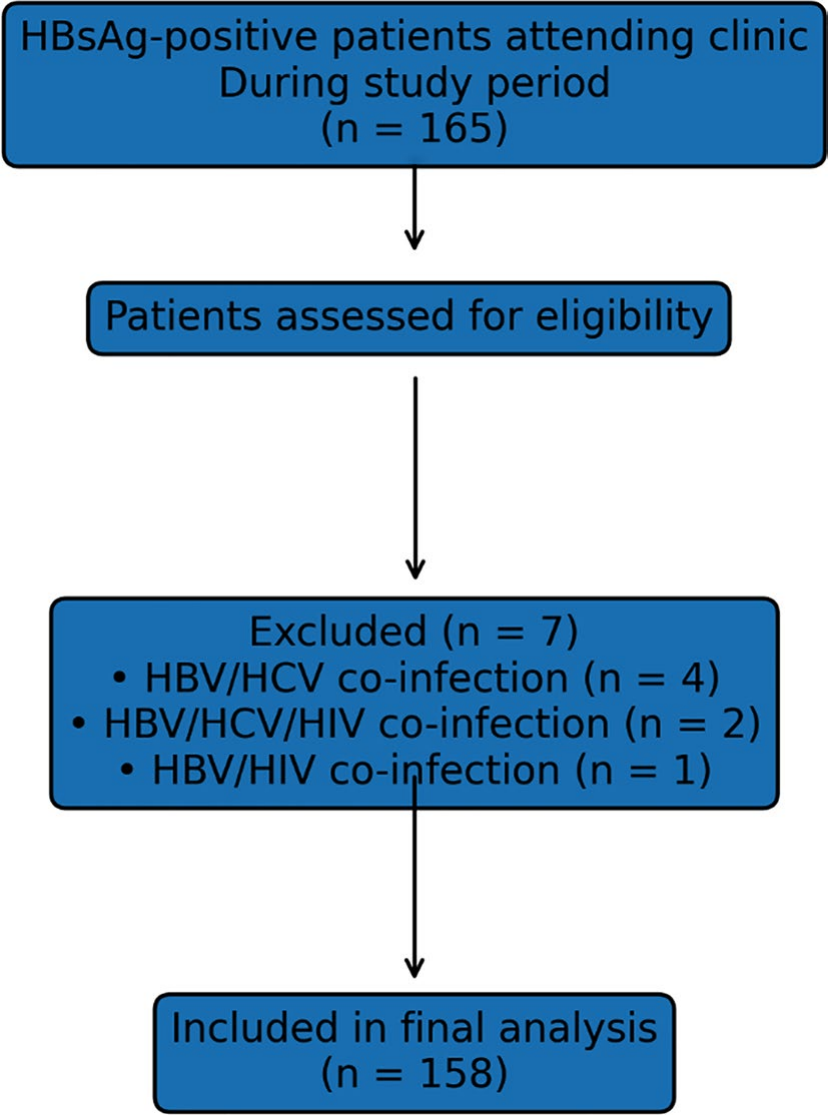
Flow chart of the study

Inclusion criteria: Adults with HBsAg positivity who attended the clinic for evaluation or routine follow-up. Patients with other combined chronic liver diseases were excluded from the study. All eligible patients were recruited consecutively till the final sample size was achieved. During the study period, 165 patients had attended the clinic. Four patients were excluded due to combined HBV/HCV infections, 2 patients were excluded due to combined HBV/HCV/HIV infections, and 1 patient was excluded due to combined HBV/HIV infections. All other patients were included (Fig. 1).

The study was approved by the ethical committee board in the Faculty of Medicine, Alexandria University. IRB NO: 00012098, serial number: 0107523. The study was conducted in accordance with the provisions of the Declaration of Helsinki and Good Clinical Practice guidelines. All patients included in the study provided informed consent.

### Methods

All HBsAg-positive participants included in the study were assessed at the time of enrollment as follows:

1. **Clinical assessment:**

This was focused on symptoms and signs of chronic liver disease (jaundice, hepatic encephalopathy, and variceal bleeding, non-specific symptoms of chronic hepatitis), liver and spleen sizes, as well as other comorbidities. Jaundice was defined as clinically evident icterus, while hepatic encephalopathy as neuropsychiatric manifestations documented by the treating hepatologist, and variceal bleeding as documented history of upper gastrointestinal bleeding attributed to varices.

2. **Laboratory investigations**,** including**:

- Complete blood picture (CBC) [13], chemical tests that included serum alanine aminotransferase (ALT), serum aspartate aminotransferase (AST), serum albumin, serum bilirubin, blood urea, serum creatinine, phosphorus, uric acid, and urinary albumin/creatinine ratio (UACR) [14], International normalized ratio [15], and serum Alpha-fetoprotein (AFP) level [16].
- **Viral testing**:

Hepatitis C virus (HCV) antibody, HBsAg, hepatitis B virus e antigen (HBeAg), and human immunodeficiency virus (HIV) antibody were performed on ARCHITECT i1000SR immunoassay automated analyzers (Abbott, USA) [17].

HBV DNA by real-time PCR: The real-time HBV PCR viral load was performed using the Artus^®^ HBV RG PCR kit (QIAGEN GmbH, QIAGEN Strasse 1, 40724 Hilden, Germany) [18].

- **Diagnosis of HDV by detecting HDV-specific total antibodies (HDV Ab) in the serum of infected patients:**

Testing for HDV Ab was performed at study enrollment, regardless of previous follow-up duration. To measure HDV Ab levels in serum, venous blood samples were collected. The serum was centrifuged for 20 min at 2000–3000 revolutions per minute after being left to clot for 10–20 min. It was then kept at -20 °C until it was time for analysis. The concentrations of serum HDV Ab were determined using double-antigen sandwich enzyme-linked immunosorbent assay (ELISA) kits supplied by Shanghai Sunred Technology (China).

An antigen specific for HDV Ab was coated on microstrip plates. Standards and samples were pipetted into the wells, and any HDV Ab present was bound by the immobilized antigen. Then a detection antigen labeled with biotin and streptavidin-horseradish peroxidase conjugate was added successively to each microplate well and incubated for 60 min. After washing, chromogen solutions A and B were added to allow color development. The reaction was terminated by the addition of stop solution, and absorbance was subsequently measured at a wavelength of 450 nm using a spectrophotometer. By comparing the samples’ optical densities to the standard curve, the concentrations in the samples were ascertained. HDV Ab kits have a sensitivity of 0.328 ng/L and a range of 0.4–100 ng/L. The intra-assay and inter-assay coefficients of variation were reported by the manufacturer to be < 10% and 12%, respectively. According to the manufacturer’s instructions, samples with anti-HDV levels ≥ 8 ng/L were considered positive. To further evaluate the appropriateness of this cutoff, data obtained from 20 independent healthy controls and 20 HDV PCR-positive patients were analyzed using change-point analysis. The optimal threshold was identified at the point of greatest distributional divergence between the two groups and corresponded closely to the mean optical density of the healthy controls plus two standard deviations [19]. Calibration curve analysis further supported the selected cutoff. All protocols followed the manufacturers’ instructions [20, 21].

3. **Child-Pugh classification of the severity of liver disease for cirrhotic patients.** [22]
4. **Imaging evaluation**:

- **Abdominal ultrasonography** [23].
- **Triphasic computed tomography (CT) and/or dynamic magnetic resonance imaging (MRI)** were done to assess the characteristics of focal hepatic lesions if present [24, 25].
- **Transient elastography (TE)** (FibroScan^®^, Echosens, Paris, France) was used for the measurement of liver stiffness, used to assess liver fibrosis, expressed in kilopascals (kPa). At least ten validated measures and an interquartile range (IQR) that represents differences between liver stiffness measurements within 30% of the median value were necessitated for optimal quality. F0-F1 corresponds to absent or minimal fibrosis; ≥F2 corresponds to significant fibrosis; ≥F3 corresponds to advanced fibrosis; and F4 corresponds to cirrhosis. The liver stiffness cut-off was 7.25 kPa for the diagnosis of significant fibrosis (F ≥ 2), 9.4 kPa for the diagnosis of advanced fibrosis, and 12.4 kPa for the diagnosis of cirrhosis [26, 27].

5. **The type of treatment of HBV was documented for patients who were on treatment.**

### Statistical analysis of the data

IBM SPSS software version 20.0 was used to perform the statistical analysis of the data (Armonk, NY: IBM Corp., released 2011). Numbers and percentages were used to summarize categorical data. The chi-square test was performed to compare the two groups under study. Nevertheless, the Fisher Exact test or Monte Carlo correction was used when over 20% of the cells had an anticipated count below 5. The Kolmogorov-Smirnov test was used to determine whether continuous data was normal. The range, mean, standard deviation, and median were used to display the quantitative data. The two groups were compared using the Student t-test for normally distributed quantitative variables and the Mann-Whitney test for non-normally distributed quantitative variables. All statistical tests were conducted with a significance threshold of 5%.

## Results

Patients who participated in this study were 74 males (46.8%) and 84 females (53.2%), and their ages ranged between 20 and 72 years, with a mean ± SD of 46.41 ± 11.87 years.

At the beginning of the study, 114 patients (72.2%) had never received any treatment for HBV, from whom 45 patients received entecavir, 33 patients received tenofovir, and 36 patients were not indicated for treatment after recruitment. On the other hand, 44 patients (27.8%) were on treatment for HBV; 30 patients were on entecavir, and the remaining were on tenofovir.

All patients enrolled in the study were HBsAg positive. Regarding the level of viremia of HBV, 36 patients (22.8%) had undetectable levels of HBV DNA; all of them were on treatment for HBV. On the other hand, 122 patients (77.2%) were positive for HBV DNA; 114 patients were naïve, and the remaining patients were on treatment for HBV; all of them have been treated for less than one year and achieved a partial response. HBV DNA values ranged between 0.0 and 170,000 × 103 IU/ml, with a median (IQR) of 0.36 (0.02–19.0) x103 IU/ml.

Regarding transient elastography assessment, 70 patients (44.3%) had F0-F1, while 88 patients (55.7%) had significant fibrosis (F ≥ 2): 55 patients (34.8%) had F2; 3 patients (1.9%) had F3; and 30 patients (19%) had F4. And from all patients enrolled in the study, 88 patients (55.7%) were S0, 43 patients (27.2%) were S1, 25 patients (15.8%) were S2, and 2 patients (1.3%) were S3.

Out of the 30 cirrhotic patients (19.0%) based on transient elastography assessment, 19 patients (63.3%) were Child-Pugh class A, while 9 participants (30.0%) were Child-Pugh class B, and only 2 patients (6.7%) were Child-Pugh class C.

From all patients enrolled in the study, 31 patients (19.6%; 95% CI, 13.4%-26.8%) were positive for HDV total antibodies, while the remaining patients (80.4%) were negative.

Age was significantly higher among HDV antibody-positive patients (Table 1). Regarding recorded clinical parameters, there was no statistically significant discrepancy between the two groups except for the presence of hepatic encephalopathy (*p* = 0.014). (Table 1) Regarding laboratory parameters, the median serum levels of ALT and AST were statistically higher among patients positive for the HDV antibody (*p* = 0.010 and 0.009, respectively). Also, the median of HBV DNA levels was statistically lower among this group (*p* = 0.017) (Table 1).

**Table 1:** Comparison between anti-hepatitis D negative and anti-hepatitis D positive patients according to demographic, clinical, and laboratory parameters (*n* = 158)

	Hepatitis D virus antibody		Test of Sig.	*p*
	Positive (*n* = 31)	Negative (*n* = 127)		
**Gender**				
Male	14 (45.2%)	60 (47.2%)	χ^2^ = 0.043	0.835
Female	17 (54.8%)	67 (52.8%)		
**Age (years)**	50.90 ± 11.97	45.31 ± 11.63	t = 2.343^*^	0.024^*^
**Arthralgia**	3 (9.7%)	11 (8.7%)	χ^2^ = 0.032	^FE^*p*=1.000
**Upper gastrointestinal variceal bleeding**	4 (12.9%)	8 (6.3%)	χ^2^ = 1.548	^FE^*p*=0.253
**Hepatic encephalopathy**	4 (12.9%)	2 (1.6%)	χ^2^ = 8.753^*^	^FE^*p*=0.014^*^
**Jaundice**	3 (9.7%)	4 (3.1%)	χ^2^ = 2.508	^FE^*p*=0.137
**Treatment status at the beginning of the study**				
Naïve	22 (71%)	92 (72.4%)	χ^2^ = 0.027	1.000
Treated	9 (29%)	35 (27.6%)		
**Treatment status after recruitment**				
Not currently on antiviral treatment	9 (29%)	27 (21.3%)	χ^2^ = 3.506	0.173
Entecavir	17 (54.8%)	58 (45.7%)		
Tenofovir	5 (16.1%)	42 (33.1%)		
**Hemoglobin concentration (g/dL)**	11.89 ± 2.17	12.57 ± 1.88	t = 1.754	0.081
**Platelets (10** ^**3**^ **/µl)**	177.0 (27.0–460.0)	219.0 (20.0–484.0)	U = 1530.0	0.055
**Alanine transaminase (U/L)**	38.0 (19.0–164.0)	32.0 (9.0–125.0)	U = 1379.0^*^	0.010^*^
**Aspartate transaminase (U/L)**	40.0 (15.0–255.0)	31.0 (11.0–323.0)	U = 1374.5^*^	0.009^*^
**Serum albumin (g/dL)**	4.05 ± 0.67	4.15 ± 0.51	t = 0.766	0.448
**Serum bilirubin (mg/dL)**	0.60 (0.20–3.30)	0.50 (0.20–3.40)	U = 1802.0	0.462
**International normalized ratio (INR)**	1.11 ± 0.22	1.09 ± 0.19	t = 0.599	0.550
**Alpha Fetoprotein (ng/ml)**	4.10 (1.60–60.0)	4.0 (1.20–1000.0)	U = 1910.5	0.799
**Hepatitis B virus e antigen (HBeAg)**	3 (9.7%)	21 (16.5%)	χ^2^ = 0.910	^FE^*p*=0.416
**Median Hepatitis B virus DNA ** **(x10**^**3**^ **IU/ml)**	22 (71.0%)	100 (78.7%)	χ^2^ = 0.856	0.355
	0.04 (IQR = 33-8630) [0.00–77700]	0.70 (IQR = 110-240000) [0.00–170000]	U = 1425^*^	0.017^*^

Normally quantitative data was expressed in **mean ± SD**

Abnormally distributed data was expressed in **median (min. - max.)**

**t: Student t-test**, **U: Mann-Whitney test**, χ^2^: Chi-square test, **FE: Fisher Exact test**

p: p value for comparing between negative and positive       *: Statistically significant at p ≤ 0.05

Among patients positive for the HDV antibody, 23 patients (74.2%) had significant fibrosis (F ≥ 2). The difference was significantly higher than the negative HDV antibody group (*p* = 0.021) (Table 2).

The presence of ascites and HCC was higher among the group positive for the HDV antibody, but the difference was not statistically significant. (*p* = 0.21 & 0.25, respectively) (Table 2).

**Table 2:** Comparison between anti-hepatitis D negative and anti-hepatitis D positive patients according to imaging parameters (*n* = 158)

	Hepatitis D virus antibody		χ^2^	*P*
	Positive (*n* = 31)	Negative (*n* = 127)		
**Liver cirrhosis**	9 (29.0%)	21 (16.5%)	χ^2^ = 2.530	0.112
**Portal vein diameter**				
Average	26 (83.9%)	110 (86.6%)	0.156	^FE^p= 0.772
Dilated	5 (16.1%)	17 (13.4%)		
**Portal vein patency**				
Patent	30 (96.8%)	127 (100.0%)	4.123	^FE^p= 0.196
Thrombus	1 (3.2%)	0 (0.0%)		
**Ascites**				
No	25 (80.6%)	115 (90.6%)	4.150	^MC^p= 0.210
Mild	5 (16.1%)	8 (6.3%)		
Moderate	0 (0.0%)	2 (1.6%)		
Marked	1 (3.2%)	2 (1.6%)		
**Hepatocellular carcinoma**	2 (6.5%)	3 (2.4%)	1.360	^FE^p= 0.254
**Fibrosis degree**				
F0-F1	8 (25.8%)	62 (48.8%)	5.348^*^	0.021^*^
F2 – F4	23 (74.2%)	65 (51.2%)		

Qualitative data were described using number and percent

χ^2^: Chi-square test, FE: Fisher Exact test, MC: Monte Carlo test

p: p-value for comparing between negative and positive, *: Statistically significant at p ≤ 0.05

We performed a univariate and multivariate logistic regression analyses to evaluate factors associated with significant fibrosis. The univariate analysis included HDV antibody status, age, HBV DNA level, and antiviral treatment status as clinically relevant covariates. Variables that were statistically significant in the univariate analysis were subsequently entered into a multivariate logistic regression model. This analysis demonstrated the independent association between HDV positivity and the presence of significant fibrosis (Tables 3 and 4).

**Table 3:** Univariate logistic regression analysis for the factors associated with significant fibrosis (*n* = 88) vs. no fibrosis (*n* = 70)

	Univariate analysis	
	*P*	OR (95%C.I)
Age (years)	0.041^*^	1.029 (1.001–1.057)
Treatment status at the beginning of the study		
Naïve ^®^	1.000	–
Treated	0.860	1.065 (0.528–2.148)
Hepatitis D virus antibody		
Negative ^®^	1.000	–
Positive	0.024^*^	2.742 (1.141–6.588)
HBV DNA (log 10 IU/mL)	0.994	0.91 (0.76–1.1)

OR: Odds ratio, C.I: Confidence interval

*: Statistically significant at p ≤ 0.05

®: reference type

**Table 4:** Multivariate logistic regression analysis of factors independently associated with significant fibrosis (*n* = 88) vs. no fibrosis (*n* = 70)

	Multivariate analysis	
	*P*	OR (95%C.I)
Age (years)	0.092	1.024 (0.996–1.053)
Hepatitis D virus antibody		
Negative ^®^	1.000	–
Positive	0.049^*^	2.442 (1.002–5.952)

OR: Odds ratio, C.I: Confidence interval

*: Statistically significant at p ≤ 0.05

®: reference type

## Discussion

Our study, to our knowledge, is the first to report HDV seroprevalence in Alexandria, revealing a rate of 19.6%. Chronic HDV infection had been identified as the most serious type of chronic viral hepatitis in human beings, resulting in accelerated progression of liver disease. People chronically infected with HDV have a greater risk of cirrhosis, hepatic decompensation, HCC, LT, and liver-related mortality [28–30]. These make HDV an important yet frequently underdiagnosed contributor to advanced liver disease.

According to the EASL as well as the APASL guidelines, screening for HDV is advised in all HBsAg-positive patients [31, 32]. In contrast, the AASLD guidelines advocate for HDV screening only in HBsAg-positive persons at risk, and this was not changed in the last guidelines updates [33]. Emerging evidence, however, revealed that risk-based screening failed to detect a significant proportion of HDV infections, and the implementation of reflex testing for HDV antibodies among all HBsAg-positive persons resulted in a 5-fold increase in detection of HDV infections, facilitating a more precise assessment of disease burden [34]. Moreover, only a small fraction of HBsAg-positive persons are actually screened for HDV, and the actual number of individuals infected with HDV is therefore underestimated [35].

In Egypt, data about the prevalence of HDV infection among HBV-infected patients is scanty and heterogeneous. Egypt currently does not implement universal HDV screening among HBV-infected patients, and testing depends only on clinician discretion. Confirmatory PCR for HDV RNA is available only at private laboratories. No HDV-directed therapy is available in Egypt. This makes assessing the magnitude of the problem important to help policymakers in their decisions.

Earlier studies from Egypt reported heterogeneous results ranging from 3.4% to as high as 47.7%. This marked variability likely resulted from differences in study population (acute versus chronic HBV, inactive carriers, referral-based cohorts), geographic differences, time periods, and diagnostic assays [12, 36–41]. Supplementary table S1 summarizes the studies on Egyptian HDV prevalence. Globally and in line with our results, Stockdale et al. reported that HDV prevalence among HBsAg-positive individuals visiting hepatology clinics was 16.4%, although the estimated global prevalence was 4.5% in all HBsAg-positive individuals [9]. The differences between studies underscore the importance of regional data in defining the true magnitude of the problem.

In the present study, HDV seroprevalence was not associated with patient sex, a finding consistent with several studies [39, 40]. In contrast, HDV-positive patients were significantly older than HDV-negative ones, which could be due to the cumulative exposure to risk factors for HDV over time. Many other studies from Egypt and Africa reported the same finding [38, 41, 42] On the other hand, Papatheodoridis et al. reported higher HDV prevalence among younger individuals. They attributed this to sexual transmission and intravenous drug use in younger age groups [43]. This suggests different transmission dynamics among different regions. In developed countries, patterns of transmission are evolving as a result of proper sterilization of equipment and availability of disposable medical products. Prevalence, and therefore transmission, remains higher amongst PWID, MSM, and sex workers [44].

Our study found that liver enzymes were significantly elevated in anti-HDV-positive patients, suggesting more liver necroinflammation. This finding is consistent with the known HDV effects and with the results of many other previous studies [38, 45, 46]. On the other hand, Makhlouf et al., as well as Alizadeh et al., showed that there was no statistically significant discrepancy between the two groups as regards the mean values of ALT and AST. In the former study, this could be attributed to the presence of a higher percentage of acute hepatitis B in the anti-HDV negative group, while in the later study, this could be justified by the small sample size [41, 47].

A key finding of this study is the significantly higher prevalence of significant fibrosis among anti-HDV-positive patients. Nearly three-quarters of anti-HDV-positive patients had significant fibrosis (F ≥ 2) in comparison to about half of anti-HDV-negative patients. In multivariate logistic regression analysis, HDV antibody-positivity remained independently associated with significant fibrosis. Anti-HDV-positive patients had approximately 2.4-fold higher odds of significant fibrosis compared with anti-HDV-negative patients. This observation supports the well-known role of HDV in enhancing the fibrosis progression [2, 3]. Similarly, other studies reported the association between HDV infection and the higher prevalence of liver fibrosis, reinforcing the robustness of our finding [48, 49].

With regard to clinical liver decompensation, hepatic encephalopathy was significantly more frequent among anti-HDV-positive patients, a finding compatible with HDV’s known propensity for rapid decompensation [2, 3]. In contrast, no significant differences were observed in our cohort regarding the development of ascites or variceal bleeding. But these findings should be interpreted cautiously, as other factors like disease duration, treatment status, competing risk factors, and modest cohort size can influence these findings. Other studies also show higher risk of liver decompensation in the form of hepatic encephalopathy and/or ascites development among HDV-positive patients [48, 50, 51] while Ananchuensook et al. showed no significant discrepancy between both groups as regards the occurrence of variceal bleeding [52].

Our study demonstrated that there was a higher incidence of HCC among those with HDV seropositivity, but the difference was not statistically significant. This may reflect the limited sample size to detect a significant difference and the multifactorial nature of hepatocarcinogenesis. Nevertheless, previous studies have clearly demonstrated an increased risk of HCC in HDV-infected patients [53, 54]. This suggests that continued long-term follow-up is necessary.

Consistent with previous observations, levels of HBD DNA were significantly lower in anti-HDV-positive patients, suggesting a suppressive effect of HDV on HBV replication. This phenomenon has been described across other studies [35, 55]. The coexistence of lower HBV viral loads and more significant fibrosis highlights the direct pathogenic effect of HDV on liver disease progression.

The high HDV seroprevalence along with markers of more advanced liver disease strongly support the need for universal HDV screening among all HBsAg-positive individuals in Egypt, in line with EASL recommendations [30]. Reliance on risk-based screening is likely to miss a substantial proportion of HDV cases who are at greater risk for adverse outcomes. Early identification of HDV infection is particularly important in the current era of emerging HDV-directed therapeutic options [30, 56].

The strengths of our study include its prospective design, detailed clinical assessment, and transient elastography-based fibrosis staging in line with recent clinical practice guidelines. However, several limitations should be acknowledged. The single-center setting may limit generalizability, while the modest sample size restricted the ability to detect differences in less frequent clinical outcomes. Duration since HBV diagnosis and duration of treatment were not consistently available for all participants and therefore were not included in the analysis. Additionally, the reliance on total anti-HDV antibodies without PCR confirmation precludes differentiation between active and resolved infections. Future multicenter studies incorporating HDV RNA assessment are warranted to better identify the true burden and clinical impact of HDV infection in the different geographic areas of Egypt.

## Conclusions

Seroprevalence of HDV is common among HBV-infected patients in Alexandria. It is associated with higher disease activity, more advanced liver fibrosis, and increased risk of hepatic decompensation. This should highlight HDV as a significant yet underrecognized contributor to liver disease severity in Egypt. Integrating universal HDV screening into the national HBV programs and conducting multicenter studies are crucial to accurately define the national disease burden, inform national policy decisions, and guide future management strategies in the era of emerging HDV-directed therapies.

## Supplementary Information

Below is the link to the electronic supplementary material.

Supplementary Material 1

## Data Availability

All datasets generated and analysed during the current study are available on request from the corresponding author.

## Abbreviations

AASLD: American Association for the Study of Liver Diseases
AFP: Alpha fetoprotein
ALT: Alanine aminotransferase
HDV Ab: HDV antibodies
APASL: Asian-Pacific Association for the Study of the Liver
AST: Aspartate aminotransferase
CBC: Complete blood picture
CHD: Chronic hepatitis D
CT: Computed tomography
EASL: European Association for the Study of the Liver
ELISA: Enzyme-linked immunosorbent assay
HBeAg: Hepatitis B virus e antigen
HBsAg: Hepatitis B surface antigen
HBV: Hepatitis B virus
HCC: Hepatocellular carcinoma
HCV: Hepatitis C virus
HDV: Hepatitis D virus
HIV: Human immunodeficiency virus
INR: International normalized ratio
IQR: Interquartile range
kPa: Kilopascals
MRI: Magnetic resonance imaging
MSM: Men who have sex with men
PWID: People who inject drugs
TE: Transient elastography
UACR: Urinary albumin/creatinine ratio
