# Supplementary material for: Seroprevalence of hepatitis D among hepatitis B-infected patients attending the viral hepatitis treatment unit in Alexandria, Egypt: a prospective cross-sectional observational study
Source: BMC Infect Dis. 2026 Aug 14;26:1507. doi: 10.1186/s12879-026-13956-6 (PMC13483714; doi:10.1186/s12879-026-13956-6)
Supplement: Supplementary file 1 [file 12879_2026_13956_MOESM1_ESM.docx]

**Supplementary Material:**

**Table S1: Summary of Egyptian HDV prevalence studies**

| **Study (Year)** | **Setting / Location** | **Population** | **Sample Size** | **Detection Method** | **Prevalence (%)** |
| --- | --- | --- | --- | --- | --- |
| El Zayadi et al., 1988 (12) | Cairo | Chronic HBV patients | 44 | Anti-HDV (total) | 47.7 |
| El Zayadi et al., 1988 (12) | Cairo | Inactive HBsAg carriers | 48 | Anti-HDV (total) | 8.3 |
| Darwish et al., 1992 (36) | Egypt (multi-site) | Acute HBV | 124 | Anti-HDV | 16.9 |
| Darwish et al., 1992 (36) | Egypt (multi-site) | Chronic HBV | 51 | Anti-HDV | 23.5 |
| Darwish et al., 1992 (36) | Egypt (multi-site) | Inactive carriers | 41 | Anti-HDV | 21.9 |
| Zaki et al., 2010 (37) | Cairo | HBsAg-positive | 100 | Anti-HDV | 20.0 |
| Ahmed et al., 2016 (38) | Cairo, Beni-Suef | HBsAg-positive | 216 | Anti-HDV (total) | 14.8 |
| Fouad et al., 2016 (39) | Cairo | HBsAg-positive | 121 | Anti-HDV IgG | 8.3 |
| Elzefzafy et al., 2022 (40) | Nile Delta | HBsAg-positive | 631 | Anti-HDV IgG | 3.4 |
| Makhlouf et al., 2019 (41) | Upper Egypt | HBsAg-positive | 186 | Anti-HDV (total) | 43.0 |
| **Current study (2025)** | Alexandria | HBsAg-positive | 158 | Anti-HDV (total) | **19.6** |
